# 

**Published:** 1903-08

**Authors:** 


### CONTENTS.

ORIGINAL COMMUNICATIONS—

THE PREVENTION OF DIARRHEAL DISEASES IN INFANTS. By Robert W. Hynds, M.D., Atlanta, Ga...... 289

REPORT OF INTERESTING OPERATIVE CASES AT PRESBYTERIAN HOSPITAL. By E. C. Davis, M.D., Atlanta, Ga...... 295

THE ACTION ON MUCOUS MEMBRANES OF SILVER SALTS, WITH ESPECIAL REFERENCE TO SOME OF THEIR NEW ORGANIC FORMS. By Arthur G. Hobbs, M.D., Atlanta, Ga...... 300

PHRENOLOGY AND LIFE VOCATIONS. By E. G. Ferguson, M.D., Macon, Ga...... 304

SOCIETY REPORTS—

CLINICAL SOCIETY OF THE NEW YORK POLYCLINICAL MEDICAL SCHOOL AND HOSPITAL...... 308

EDITORIAL NOTES AND COMMENTS—

THE FROUDE-CARLYLE CONTROVERSY...... 315

THE "CHRISTIAN HOSPITAL"...... 316

NEWS AND NOTES...... 318

CONTENTS CONTINUED...... X

